# Soluble immune checkpoints as correlates for HIV persistence and T cell function in people with HIV on antiretroviral therapy

**DOI:** 10.3389/fimmu.2023.1123342

**Published:** 2023-03-28

**Authors:** Chris Y. Chiu, Maya D. Schou, James H. McMahon, Steven G. Deeks, Rémi Fromentin, Nicolas Chomont, Michelle N. Wykes, Thomas A. Rasmussen, Sharon R. Lewin

**Affiliations:** ^1^ Department of Infectious Diseases, The University of Melbourne at The Peter Doherty Institute for Infection and Immunity, Melbourne, VIC, Australia; ^2^ Department of Infectious Diseases, Alfred Hospital and Monash University and the Alfred Hospital, Melbourne, VIC, Australia; ^3^ Department of Medicine, University California San Francisco, San Francisco, CA, United States; ^4^ Centre de Recherche du Centre Hospitalier de l’Université de Montréal, Montreal, QC, Canada; ^5^ Department of Microbiology, Infectiology and Immunology, Faculty of Medicine, Université de Montréal, Montreal, QC, Canada; ^6^ QIMR Berghofer Medical Research Institute, Brisbane, QLD, Australia; ^7^ Department of Infectious Diseases, Aarhus University Hospital, Aarhus, Denmark; ^8^ Victorian Infectious Diseases Service, Royal Melbourne Hospital at the Peter Doherty Institute for Infection and Immunity, Melbourne, VIC, Australia

**Keywords:** HIV, soluble immune checkpoint, immunotherapy, immune checkpoint blockade, HIV reservoir, HIV-specific T-cell function

## Abstract

**Introduction:**

In people with HIV (PWH) both off and on antiretroviral therapy (ART), the expression of immune checkpoint (IC) proteins is elevated on the surface of total and HIV-specific T-cells, indicating T-cell exhaustion. Soluble IC proteins and their ligands can also be detected in plasma, but have not been systematically examined in PWH. Since T-cell exhaustion is associated with HIV persistence on ART, we aimed to determine if soluble IC proteins and their ligands also correlated with the size of the HIV reservoir and HIV-specific T-cell function.

**Methods:**

We used multiplex bead-based immunoassay to quantify soluble programmed cell death protein 1 (PD-1), cytotoxic T-lymphocyte-associated protein 4 (CTLA-4), lymphocyte activation gene-3 (LAG-3), T cell immunoglobulin domain and mucin domain 3 (TIM-3), PD-1 Ligand 1 (PD-L1) and PD-1 Ligand 2 (PD-L2) in plasma from PWH off ART (n=20), on suppressive ART (n=75) and uninfected controls (n=20). We also quantified expression of membrane-bound IC and frequencies of functional T-cells to Gag and Nef peptide stimulation on CD4+ and CD8+ T-cells using flow cytometry. The HIV reservoir was quantified in circulating CD4+ T-cells using qPCR for total and integrated HIV DNA, cell-associated unspliced HIV RNA and 2LTR circles.

**Results:**

Soluble (s) PD-L2 level was higher in PWH off and on ART compared to uninfected controls. Higher levels of sPD-L2 correlated with lower levels of HIV total DNA and higher frequencies of gag-specific CD8+ T-cells expressing CD107a, IFNγ or TNFα. In contrast, the concentration of sLAG-3 was similar in uninfected individuals and PWH on ART, but was significantly elevated in PWH off ART. Higher levels of sLAG-3 correlated with higher levels of HIV total and integrated DNA, and lower frequency of gag-specific CD4+ T cells expressing CD107a. Similar to sLAG-3, levels of sPD-1 were elevated in PWH off ART and normalized in PWH on ART. sPD-1 was positively correlated with the frequency of gag-specific CD4+ T cells expressing TNF-a and the expression of membrane-bound PD-1 on total CD8+ T-cells in PWH on ART.

**Discussion:**

Plasma soluble IC proteins and their ligands correlate with markers of the HIV reservoir and HIV-specific T-cell function and should be investigated further in in large population-based studies of the HIV reservoir or cure interventions in PWH on ART.

## 1 Introduction

Antiretroviral therapy (ART) in people with HIV (PWH) has dramatically reduced HIV-related morbidity and mortality, however lifelong ART is required due to the persistence of long lived and proliferating latently infected cells (1, 2). PWH on ART also exhibit persistent immune dysfunction, including elevated expression of multiple immune checkpoint (IC) proteins on both total and HIV-specific T-cells (3–7). One approach to eliminating HIV persistence on ART is to enhance HIV-specific immunity through reversing T-cell exhaustion [reviewed in (2, 8)]. A simpler high throughput plasma-based biomarker that quantifies T-cell exhaustion could be of benefit for large population based studies of the HIV reservoir and/or HIV cure interventions. Here, we investigated whether the soluble forms of ICs correlated with their membrane bound expression, markers of the HIV reservoir, HIV-specific T-cell immunity and/or T-cell activation.

Soluble IC are generally derived from the translation of alternatively spliced mRNA that lack the exons for the transmembrane domain. This leads to generation of several soluble ICs, including soluble cytotoxic T-lymphocyte-associated protein 4 (sCTLA4) (9), programmed death -1 (sPD-1) (10), T cell immunoglobulin domain and mucin domain 3 (sTIM-3) (11), lymphocyte activation gene-3 (sLAG-3) (12), programmed cell death protein ligand 1 (sPD-L1) (13) and sPD-L2 (14). However, some soluble ICs can also be derived through other pathways. This includes the shedding or cleavage of membrane bound IC by transmembrane proteases such as Matrix Metalloproteinases (MMP) and A Disintegrin and Metalloproteinase (ADAM) expressed on the cell. Inhibitors to MMP, ADAM10 and ADAM17 have been shown to decrease the levels of membrane-bound PD-L1 (15), TIM-3 (16), LAG-3 (17) and PD-L2 (18), but not PD-1 or CTLA-4. In addition, antigen stimulation to immune cells can trigger cleavage of membrane-bound IC by transmembrane metalloproteinases and alter the pattern of alternatively spliced mRNA for sIC as has been demonstrated with sLAG-3 and sTIM-3 (17) (19). Finally, soluble ICs can also be detected in the form of exosomes which retain the intact transmembrane domain of PD-1 (20), CTLA-4 (21), TIM-3 (22), PD-L1 (23) and PD-L2 (24).

In PWH off ART compared to uninfected individuals, prior work has demonstrated elevated levels of sPD-1 and sTIM-3 in primary HIV infection (25) and elevated sPD-L1 in chronic infection (26, 27), consistent with prior findings of elevated membrane bound ICs in PWH (4, 6, 28, 29). Levels of sTIM-3 were positively correlated with HIV viral load and inversely correlated with CD4 counts in PWH off ART (16). Furthermore, in a prospective study, sPD-1, and sTIM-3 decreased on ART and correlated with membrane bound expression of the same ICs (25). To date, a systematic examination of all soluble immune checkpoints in PWH on and off ART has not been performed.

We hypothesized that in PWH treated during chronic infection and on ART, soluble IC would correlate with levels of membrane bound ICs and would also be correlated with the size of the HIV reservoir and the frequency of HIV-specific T cells. The overall goal of the study was to determine if soluble IC proteins could serve as a simpler high throughput marker of the reservoir and HIV-specific T-cell function.

## 2 Methods

### 2.1 Study subjects

Inclusion criteria were being an adult (>18 years) with HIV and either naïve to ART (off ART) or on suppressive ART (on ART) for at least three years. Clinical characteristics of all participants are summarized in **Table S1**. Off ART participants and age-matched uninfected individuals were recruited from the Observational study of the consequence of the Protease Inhibitor Era (SCOPE) cohort (NCT: NCT00187512), an observational, prospective study of PWH at the San Francisco General Hospital and University of California San Francisco (UCSF), California. PWH on suppressive ART for at least three years were recruited in San Francisco [as part of the SCOPE study and previously described in (30)] or in Melbourne, Australia (Alfred Hospital and Melbourne Sexual Health Centre) using baseline samples from participants in the Dolutegravir impact on residual replication (DIORR) study (NCT: NCT02500446), a randomized, placebo-controlled, double-blind trial of PWH receiving combination ART for at least 3 years (31). Use of all samples for this study was approved by the ethics committees at UCSF, Alfred Hospital and University of Melbourne.

### 2.2 Measurement of soluble immune checkpoint proteins

We used a custom ProcartaPlex 6-plex panel (ThermoFisher Cat # PPX-06) to detect soluble PD-1, LAG-3, TIM-3, CTLA-4, PD-L1 and PD-L2 in a 96-well plate format. The assay was performed according to manufacturer’s instruction. Soluble TIGIT was not included as this was not available as an analyte in the panel. Briefly, 4-fold serial dilution of the assay standard was performed, followed by washing magnetic beads with the supplied washing buffer. The reaction volume of 50 µL consisted of 25 µL of plasma or assay standards and 25 µL of Universal Assay Buffer. These solutions were added to wells and incubated for 120 minutes. After two washes, 25 µL of Detection Antibody Mixture was added to each well for 30 minutes on a shaker at 500 rpm. After two washes, 50 µL of Streptavidin-PE was added to each well for 30 minutes on a shaker at 500 rpm. After two washes, 120 µL of Reading Buffer was added to each well for 5 minutes on a shaker at 500rpm. All incubation steps were performed at ambient temperature. The levels of soluble IC were measured by MAGPIX (Millipore, MA, USA). Five parameter log-logistic model was used to generate a standard curve for each analyte using the R package beadplexr (32).

### 2.3 Expression of membrane bound IC and cellular activation markers

Expression of membrane bound IC and cellular activation markers in CD4+ and CD8+ T cells were measured by flow cytometry with cryopreserved peripheral blood mononuclear cells (PBMC) from participants on ART (SCOPE) (**Table 1**, **Supplementary Table 1**) as previously described (30). In brief, cryopreserved PBMC were thawed and stained with surface phenotypic markers (CD3 [Clone UCHT1], CD4 [clone S3.5, Invitrogen], CD8 [Clone RPA-T8], CD14 [Clone M5E2], CD19 [Clone SJ25C1], CD45RA [HI100], CD27 [Clone O323], CCR7 [Clone 3D12]) and immune checkpoints (PD-1 [Clone EH12.1], CTLA-4 [Clone BNI3], LAG-3 [Clone FAB2319F, R&D], TIGIT [Clone MBSA43, eBioscience], TIM-3 [Clone F38-2E2, BioLegend], CD160 [Clone By55, eBioscience], 2B4 [Clone C1.7]). All antibodies were purchased from BD Bioscience unless indicated otherwise. LSR II cytometer (BD Bioscience) was used for acquisition. FlowJo 9 was used for analysis.

**Table 1:** Clinical characteristics of participants.

Group	SCOPE		SCOPE - Aviremic	DIORR
HIV Status	Neg	Pos	Pos	Pos
Sex, male/total number	19/20	19/20	47/48	31/34
ART duration	NA	NA	7.8 (5.1 - 12.2)	NA
Age, years	55.0 (50.5 - 59.5)	56.0 (50.5 - 61.5)	56.5 (50.5 - 61.5)	47.0 (43.0 - 53.0)
CD4 Count, cells/µL	808.0 (628.5 - 1065.0)	362.0 (212.5 - 527.5)	683.5 (533.0 - 858.0)	676.5 (597.0 - 1010.0)

Participants were recruited through the SCOPE cohort (San Francisco) and DIORR clinical trial (baseline samples only, Melbourne). Unless otherwise stated, all data shows median (interquartile range). NA, not applicable.

### 2.4 Intracellular cytokine staining

PBMC were isolated by leukapheresis and cryopreserved prior to assessment of intracellular cytokine staining as previously described (7). In brief, cryopreserved PBMC were thawed and incubated at 37 °C and 5% CO_2_ overnight. PBMC stimulation was performed in a 96-well plate for 6 hours at 37°C with 5% CO2, of which each well contained 1 x 10^6^ PBMC, a cytokine secretion inhibitor cocktail (5 µg/mL Brefeldin A and 5 µg/mL Monensin), anti-CD107a and antiretrovirals (18 µM azidothymidine, 10 µM efavirenz and 20 µM raltegravir) to inhibit further rounds of viral replication following stimulation *ex vivo*. The cells were stimulated with either 0.4% DMSO, 2 µg/mL Gag, Nef or 1 µg/mL staphylococcal enterotoxin B (SEB;S4881, Sigma). After stimulation, cells were stained for the live/dead marker (Cat # L34957, Invitrogen), and with antibodies to the following surface markers (CD4 [Clone RPA-T4], CD14 [Clone M5E2], CD19 [Clone HIB19], CD45RA [Clone HI100] and CCR7 [Clone 3D12]) at ambient temperature in the dark for 30 minutes. After cell fixation and permeabilization, staining with antibodies to CD3 [Clone UCHT1], CD8 [Clone RPA-T8], IFNγ [Clone B27], TNFα [Clone MAb11] and IL-2 [Clone MQ1-17H12] was performed at ambient temperature in the dark for 30 minutes. After washing cells with perm/wash buffer twice, cells were fixed in 100 µL of 1% formaldehyde at ambient temperature in the dark for 15 minutes. All staining antibodies were obtained from BD Bioscience unless indicated otherwise. LSRFortessa cytometer (BD Bioscience) was used for acquisition. Anti-mouse and anti-rat compensation beads (Cat # 552843 & 552844, BD Bioscience) were used for compensation. The analysis of the cytometric data was performed with FlowJo 10.8.1.

### 2.5 Quantification of the HIV reservoir

We quantified total, integrated HIV DNA, 2-LTR circles as well as cell-associated unspliced HIV RNA in purified CD4+ T-cells from blood by real-time nested PCR as previously described (30, 31).

### 2.6 Statistical analysis

Statistical differences of soluble IC between the three participant groups were determined using unpaired non-parametric two-sample Wilcoxon test with a cut-off set for statistical significance of p < 0.05. A heatmap representing Spearman correlation coefficients was generated using R package corrplot (Version 0.92), where missing values are removed during the calculation of each pairwise correlation. Construction of multivariable regression models included soluble IC as the primary exposure and total/integrated HIV DNA as the outcome variables. Other variables that associated with outcome variables with statistical significance defined as p < 0.10 in the univariate analysis were considered as additional exposure variables. Forward stepwise construction of the linear multivariable regression model and the base model were compared using likelihood tests. R package ggplot2 (Version 3.3.6) was used for figures. RStudio Desktop (Version 2022.07.1 + 554) and R (Version 4.2.1) were used for statistical analyses.

## 3 Results

### 3.1 Levels of soluble IC in PWH off and on ART

**Figure 1:**
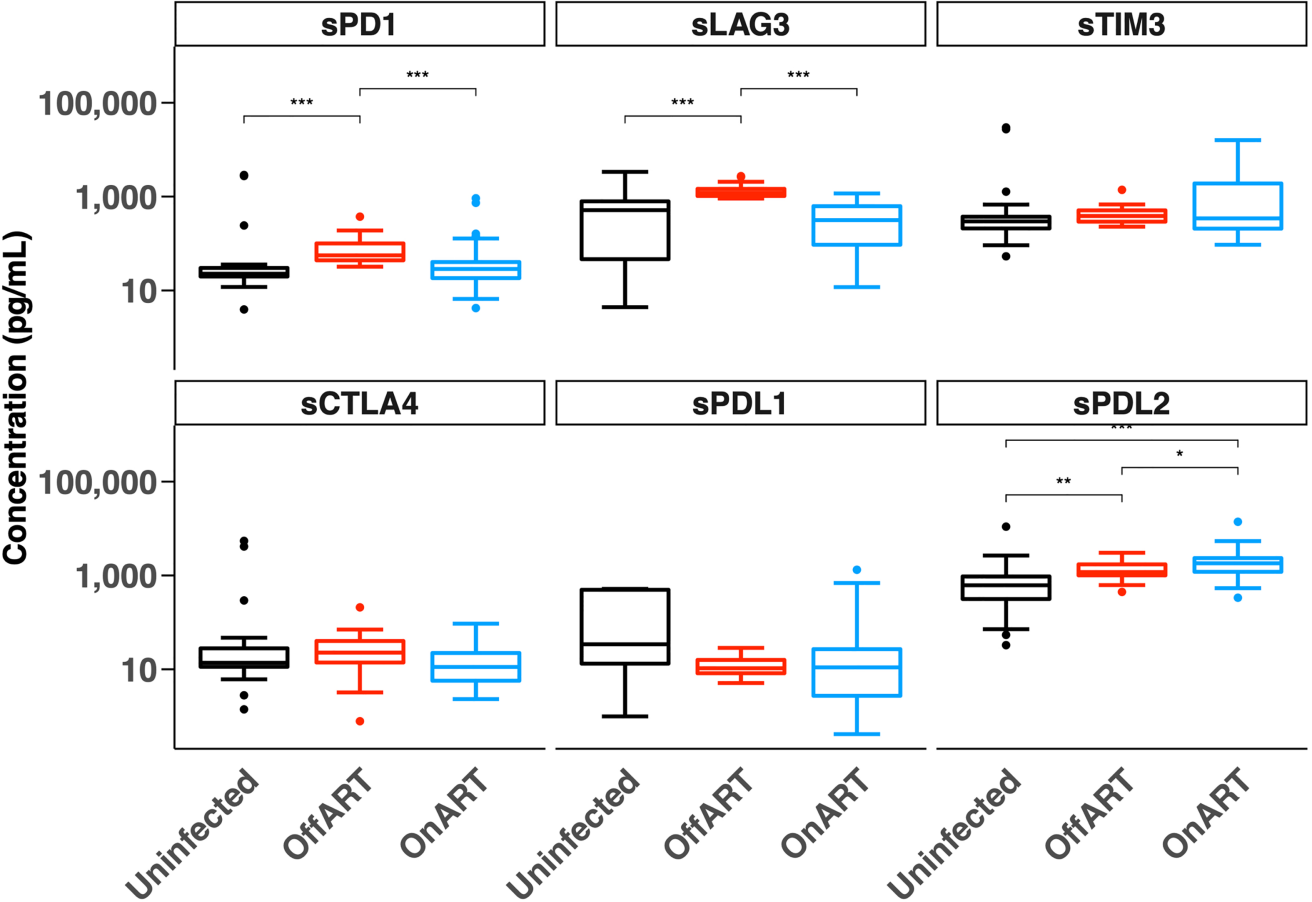
Concentration of soluble immune checkpoint proteins in HIV-uninfected participants and people with HIV off antiretroviral therapy (Off ART) and on suppressive antiretroviral therapy (On ART). Box plots show the median, 25^th^ and 75^th^ percentiles within the box and 10^th^ and 90^th^ percentiles in the error bars. The two-sample Wilcoxon test was used for comparisons between groups. *p < 0.05, **p < 0.01, ***p < 0.001. Soluble (s), Programmed Cell Death protein 1 (PD-1), Lymphocyte Activation Gene-3 (LAG-3), T cell Immunoglobulin domain and Mucin domain 3 (TIM-3), Cytotoxic T-Lymphocyte-Associated protein 4 (CTLA-4), PD-1 Ligand 1 (PD-L1) and PD-1 Ligand 2 (PD-L2).

We first quantified the concentration of six soluble ICs in plasma from three groups of participants — HIV uninfected, PWH naïve to ART (off ART) and PWH on suppressive ART (on ART) (**Figure 1**). The median levels of most soluble ICs in the off ART participants were higher than in the HIV-uninfected participants, with the difference in median levels of sLAG-3, sPD-1 and sPD-L2 reaching statistical significance (**Figure 1**). Levels of sLAG-3 and sPD-1 were significantly lower in participants on ART compared to off ART, and were similar to HIV uninfected participants. Strikingly, sPD-L2 was the only soluble protein that was elevated in participants on ART compared to both HIV uninfected and off ART participants (**Figure 1**). These results demonstrated soluble IC levels differ in PWH on and off ART and sPDL2 is significantly elevated in PWH on ART.

### 3.2 Correlations of soluble IC with clinical, immunological and virological parameters in PWH on ART

**Figure 2:**
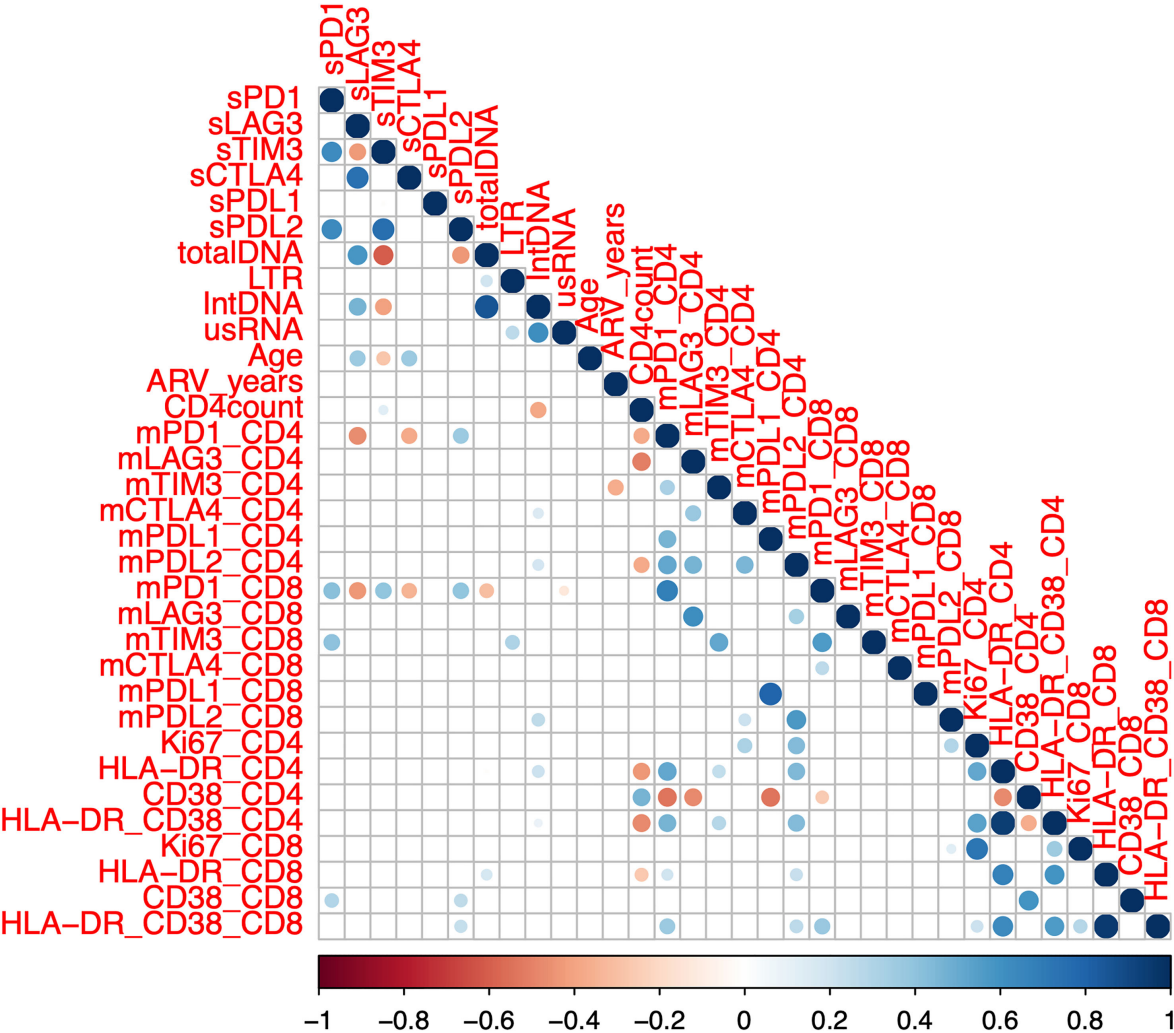
Correlation between soluble and membrane bound immune checkpoint proteins and clinical and laboratory markers in participants on antiretroviral therapy. Soluble (s) immune checkpoint proteins, reservoir size and clinical markers (n=75, SCOPE (n=48) and DIORR (n=27)), as well as membrane bound (m) immune checkpoint proteins and markers of immune activation (n = 48, SCOPE) were quantified in participants on antiretroviral therapy. Dots represent statistical significance of a given Spearman correlation with p < 0.05, and colour gradient indicates the coefficient and the directionality of the correlation. Unspliced HIV RNA (usRNA), HIV integrated DNA (intDNA), 2 LTR circles (LTR), antiretroviral duration (ARV), soluble (s) or membrane bound (m) Programmed Cell Death protein 1 (PD-1), Lymphocyte Activation Gene-3 (LAG-3), T cell Immunoglobulin domain and Mucin domain 3 (TIM-3), Cytotoxic T-Lymphocyte-Associated protein 4 (CTLA-4), PD-1 Ligand 1 (PD-L1) and PD-1 Ligand 2 (PD-L2).

Given that the overall goal of the study was to identify a simpler high throughput marker of the reservoir and HIV-specific T-cell function, we next analysed whether soluble IC correlated with these parameters in participants on ART (n=75, **Tables 1**, **2**). We assessed the correlation of soluble ICs with membrane bound ICs as well as virological, clinical, and cellular activation parameters (**Figure 2**, **Supplementary Figure 1**). The level of soluble PD-1 was positively correlated with membrane bound expression of PD-1 on CD8+ but not CD4+ T-cells (**Figure 2**, **Supplementary Figure 1**). No other soluble IC correlated with membrane bound IC.

**Table 2:** Relationship between reservoir size and soluble immune checkpoint proteins in participants on antiretroviral therapy.

	Total HIV DNA			Integrated HIV DNA		
	Estimate	Std. Error	p value	Estimate	Std. Error	p value
sPD1	-1.6578	0.9883	0.0980	-0.6464	0.5415	0.2370
sLAG3	1.4031	0.4441	0.0024	0.5546	0.2390	0.0233
sTIM3	-0.1765	0.0600	0.0044	-0.0721	0.0332	0.0331
sCTLA4	11.5560	7.0580	0.1064	1.4750	3.6470	0.6871
sPDL1	-0.1059	0.7046	0.8825	-0.0339	0.1912	0.8614
sPDL2	-0.1613	0.0765	0.0386	-0.0658	0.0419	0.1210

A univariable linear regression model was used to determine the relationship between soluble immune checkpoint proteins and total or integrated HIV DNA in participants on suppressive antiretroviral therapy (n=75, SCOPE (n=48) and DIORR (n=27)). Soluble (s) Programmed Cell Death protein 1 (PD-1), Lymphocyte Activation Gene-3 (LAG-3), T cell Immunoglobulin domain and Mucin domain 3 (TIM-3), Cytotoxic T-Lymphocyte-Associated protein 4 (CTLA-4), PD-1 Ligand 1 (PD-L1) and PD-1 Ligand 2 (PD-L2).

When we examined the relationship to reservoir size and activity, we found that higher levels of sLAG-3 correlated with higher levels of both total and integrated HIV DNA, whereas the opposite was observed for sTIM-3 and sPD-L2, where lower plasma concentrations correlated with higher levels of total HIV DNA (**Figure 2**, **Supplementary Figure 1**). Soluble TIM-3 was also negatively correlated with the levels of integrated HIV DNA (**Figure 2**, **Supplementary Figure 1**). There were no significant associations between either cell associated unspliced HIV RNA or 2LTR circles and the soluble ICs. CD4 count was weakly positively correlated with sTIM-3, contrasting to the previously reported negative correlation between sTIM-3 and CD4 count (16, 33). There was a modest positive correlation between the frequency of activated CD38 single positive or HLA-DR/CD38 double positive CD8+ T-cells and sPD-L2 (**Figure 2**, **Supplementary Figure 1**). Together, these data demonstrated that the size of the reservoir measured as either total or integrated HIV DNA correlated inversely with sTIM-3 and sPD-L2 and positively with levels of sLAG-3.

To better understand the relationships of the statistically significant correlations observed in the Spearman correlations (**Figure 2**, **Supplementary Figure 1**), we next used a linear regression model with sLAG-3, sTIM-3 and sPD-L2 as independent variables, as well as total and integrated HIV DNA as dependent variables. Using forward stepwise regression to construct a multivariable linear regression model, the base model with only the soluble IC as independent variables showed a similar fit to the observed counts of total and integrated HIV DNA. Therefore, univariable linear regression models were used for the subsequent analysis. sLAG-3 was identified as a positive predictor for total and integrated HIV DNA, where one unit (pg/mL) increase in sLAG-3 resulted in 1.40 and 0.55 increase in total and integrated HIV DNA respectively (copies per million CD4 cells, **Table 2**). In contrast, the level of sTIM-3 was a negative predictor where one unit (pg/ml) increase in sTIM-3 resulted in a reduction of 0.18 and 0.07 for total and integrated HIV DNA respectively (copies per million CD4 cells, **Table 2**). Similar to sTIM-3, a one unit (pg/mL) increase of sPD-L2 predicted a decrease in total HIV DNA of 0.16 copies per million CD4 cells (**Table 2**). In summary, the findings from the linear regression model supported correlations observed using Spearman correlations.

### 3.3 Correlates of soluble IC with HIV-specific T-cells in PWH on ART

**Figure 3:**
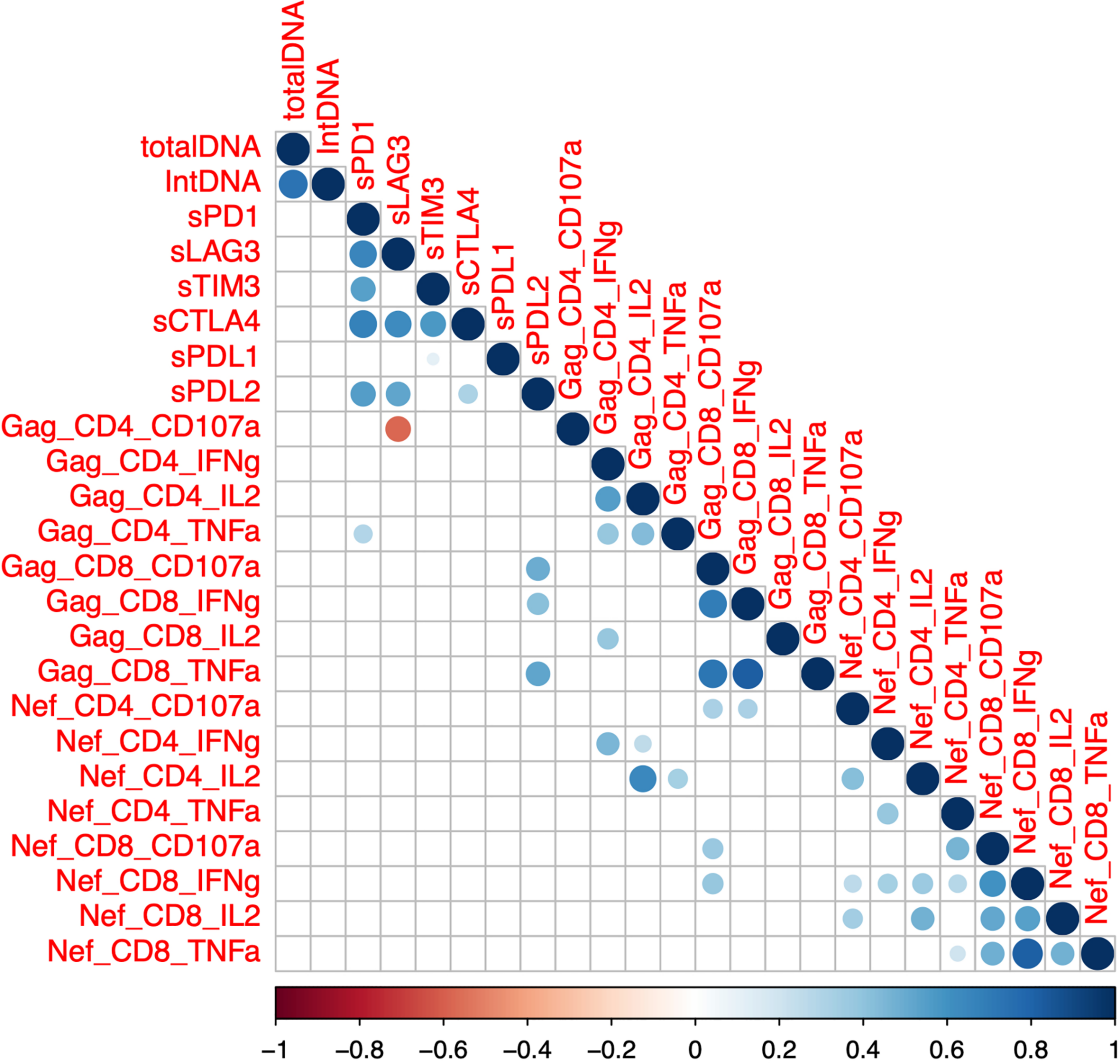
Correlation between soluble immune checkpoint proteins, reservoir size and the frequency of Gag or Nef-specific CD4+ and CD8+ T cells. Intracellular cytokine staining was performed using peripheral blood mononuclear cells from participants on antiretroviral therapy (SCOPE (n=48)) and expression of interferon-gamma (IFN-g), tumor necrosis factor-alpha (TNF-a), interleukin-2 (IL-2) and the degranulation marker CD107a was quantified using flow cytometry in both CD4 and CD8 T-cells. Soluble (s) immune checkpoint proteins were quantified in plasma as previously described. Dots represent statistical significance of a Spearman correlation with p < 0.05, and colour gradient indicates the coefficient and the directionality of the correlation. Programmed Cell Death protein 1 (PD-1), Lymphocyte Activation Gene-3 (LAG-3), T cell Immunoglobulin domain and Mucin domain 3 (TIM-3), Cytotoxic T-Lymphocyte-Associated protein 4 (CTLA-4), PD-1 Ligand 1 (PD-L1) and PD-1 Ligand 2 (PD-L2). Total DNA, total HIV DNA; intDNA, integrated HIV DNA.

We next sought to determine whether there was a correlation between soluble IC and HIV-specific T cell function in a subset of participants on ART (n=48, SCOPE, **Tables 1**, **2**). We measured the frequency of CD4+ and CD8+ T cells that produced either IFNg, TNFa, IL-2 or CD107a in response to overlapping Gag and Nef peptides. We did not observe any correlation between total and integrated HIV DNA and either gag or nef-specific T-cell responses (**Figure 3**, **Supplementary Figure 1**), in contrast to previous reports (34). Soluble PD-L2 was the only soluble IC that showed statistically significant positive correlations with the frequency of Gag-specific CD8+ T cells that express either CD107a, IFNg or TNFa (**Figure 3**, **Supplementary Figure 1**). While sLAG-3 was determined as a positive predictor for total and integrated HIV DNA (**Figure 2**; **Table 2**), sLAG-3 was negatively correlated with the frequency of Gag-specific CD4+ T-cells expressing CD107a (**Figure 3**, **Supplementary Figure 1**). sPD-1 was positively correlated with the frequency of Gag-specific CD4+ T cells expressing TNFa (**Figure 3**, **Supplementary Figure 1**). There was no significant correlations between the expression of soluble IC and IL-2. These data demonstrate a relationship between sLAG-3, sPD-L2 and sPD1 and HIV-specific T-cell functions and these relationships differed for HIV-specific CD4+ and CD8+ T-cells.

## 4 Discussion

Soluble IC can be easily measured in plasma and therefore could be a relatively simple assay to quantify changes in the HIV reservoir or HIV-specific immune function in PWH on ART. In the first comprehensive analysis of six soluble IC in PWH both off and on ART, we showed i) elevated levels of sLAG-3, sPD-1 and sPD-L2 in PWH off ART consistent with some but not all prior reports (16, 25, 35); ii) sPD-L2 was the only soluble IC that remained elevated in PWH both on and off ART; iii) HIV total and integrated DNA was predicted by the levels of several soluble ICs with a negative association with sTIM-3 and sPD-L2 and a positive association with sLAG-3; iv) Gag-specific CD4+ T-cells were associated with sLAG-3 (CD107a) and sPD-1 (TNFa) while Gag-specific CD8+ T-cells that express IFNg, TNFa and CD107a were all associated with sPD-L2.

Although the full function of soluble IC is unknown, multiple reports have demonstrated that many soluble IC retain the ability to bind their specific receptor or ligand and are bioactive (9, 13, 36–39), including sPD-1 (40), sTIM-3 (37, 38), sPD-L1 (13) and sPD-L2 (39). This has been demonstrated directly by the administration of sTIM-3 and sPD-1 ex vivo leading to an increased production of IFNg and TNFa by mice splenocytes stimulated with peptides designed for simian immunodeficiency virus (38). Presumably, sTIM-3 and sPD-1 can compete with the ligand of both ICs and will inhibit signalling through these proteins leading to enhanced T-cell function. Compared to their membrane-bound counterparts, there are contradictory reports on whether the monomeric soluble CTLA-4 and LAG-3 remain bioactive. For example, monomeric soluble CTLA-4 on its own was shown to have less binding affinity for CD80/CD86 than the dimeric membrane-bound CTLA-4 (9). However, a recent report demonstrated that repulsive guidance molecule B (RGMB)-bound soluble CTLA-4 had a higher binding affinity to CD80 than monomeric soluble CTLA-4, and could also inhibit CD80-CD28 co-stimulation on T cells (41). Finally, there are contradictory reports on whether monomeric soluble LAG-3 retains the ability to bind to MHC-II (17, 36).

No prior studies have investigated the role of sPD-L2 in PWH. First, we found that sPDL-2 was the only soluble IC that remained elevated on ART. Second, sPD-L2 was a predictor of reduced total HIV DNA as well as increased frequency of activated and functional HIV-specific CD8+ T cells. PD-L2 is expressed on multiple immune cells, including macrophages, dendritic cells and activated CD4+ and CD8+ T cells and is a ligand for PD-1, together with PD-L1 (42, 43). sPD-L2 can compete with PD-L1 for binding to PD-1, potentially resulting in activation rather than repression of the T-cell. This is similar to the formation of a PD-L1/CD80 heterodimer on antigen presenting cells that can then impair binding to CTLA-4 while retaining the capacity to bind to CD28 (44). In fact, sPDL-2 in a multimeric form has been shown to activate T-cells, i.e., it can enhance antigen-specific T-cell function *in vivo* (39). Administration of multimeric sPD-L2 in a mouse model of malaria resulted in enhanced malaria specific CD4+ T-cells and improved clinical outcomes, but similar effects to malaria-specific CD8+ T-cells were not observed (39). Finally, since correlations do not necessarily equate to causation, it is certainly possible for the higher observed level of sPD-L2 to be an effect from, rather than a cause of, more effective HIV-specific T cells. Therefore further *in vitro* studies need to be performed to address the mechanism of this relationship. However, taken together, elevated sPD-L2 was clearly associated with a smaller reservoir and enhanced HIV-specific T-cell function.

Our group has previously demonstrated that CD4+ T-cells expressing multiple ICs, including PD-1, TIGIT and LAG-3 were enriched for HIV-infected cells (30). In addition, we have shown that CD4+ T-cells expressing both PD-1 and CTLA-4 are enriched for latent HIV in PWH on ART (45, 46) and for latent SIV infection in animal models (47). We had therefore hypothesized that soluble forms of each of these ICs would correlate negatively with the size of the HIV reservoir for soluble IC that are generated by cleavage of membrane-bound IC. In this study, we did not measure soluble TIGIT, but we demonstrated a positive relationship between the HIV reservoir and sLAG3, but found no correlation between the size of the HIV reservoir and sPD1 nor sCTLA-4. It is possible that the directional differences in the relationship between the size of the HIV reservoir and membrane and soluble forms of ICs is dependent upon how the soluble ICs are generated. Membrane-bound LAG-3 can be cleaved by a surface protease following activation of the T-cell receptor leading to the production of sLAG-3 (17). In contrast, this is not a pathway shared with the formation of either sPD-1 or sCTLA-4.

Levels of sTIM-3 have been previously reported as significantly increased in PWH with primary infection and reduced following ART (25), consistent with our findings. Here, we also observed a negative correlation between sTIM-3 and reservoir size on ART. Together these studies highlight a potential role of sTIM-3 as a surrogate for virus replication as well as reservoir size. It is now well known that most virus persists on ART in a defective form (48). It will be important in future studies to examine the relationship between sTIM-3 and other soluble ICs and the frequency of cells with either intact or defective virus using the intact proviral DNA assay (48).

This study is the first comprehensive analysis of soluble IC in PWH on and off ART, however, we recognize several limitations. First, this was a cross sectional study and therefore cannot infer causality. However, our findings of lower sPD-1 and sTIM-3 in PWH on ART compared to off ART, are consistent with prior publications using matched samples (25). Future studies should examine the changes in all six soluble ICs in a prospective study of PWH following initiation of ART. Second, we used proprietary capture antibodies to soluble IC as part of a commercial bead-based multiplex assay. We cannot exclude the possibility that certain forms of soluble IC escaped detection. For example, if the epitopes targeted by the capture antibodies were in an exon which was deleted as a result of alternative splicing of mRNA or from proteolytic cleavage, they would not be detected. Third, it is possible that the detected soluble ICs were on exosomes with our method as we did not perform ultracentrifugation on the plasma samples. It is clear that ICs can persist in exosomes in studies of malignancy (20–24) and this may also be relevant in PWH. Lastly, we restricted our regression analysis on virological/immunological parameters to a single soluble IC to avoid over-fitting and over-interpretation. Using multiple soluble IC as predictors could have some advantages such as better accuracy to predict the observed reservoir size, while also accounting for potential interactions between multiple soluble IC.

In conclusion, we detected elevated levels of multiple soluble IC in plasma from PWH off ART. The levels of soluble IC declined on suppressive ART to levels similar to HIV-uninfected participants, except for sPD-L2 which remained persistently elevated. In PWH on ART, levels of soluble ICs were associated with total and integrated HIV DNA, as well as Gag-specific CD4+ and CD8+ HIV-specific T cell function. This study provides the basis for soluble IC to be further explored as a plasma based biomarker in studies aimed at understanding and/or targeting HIV persistence on ART. Further mechanistic studies on the interaction of sLAG-3, sPD-1 and sPD-L2 with both CD4+ and CD8+ T-cells from PWH may also provide novel insights into HIV persistence and immune dysfunction on ART.

## Data availability statement

The raw data supporting the conclusions of this article will be made available by the authors, without undue reservation.

## Ethics statement

The studies involving human participants were reviewed and approved by Human Ethics Advisory Group at the Alfred Hospital, University of Melbourne and University of California San Francisco. The patients/participants provided their written informed consent to participate in this study.

## Author contributions

CC and SL conceived and designed the study. CC and MS performed experiments. JM and SD coordinated clinical sample collection and provided clinical samples. CC, MS, and TR performed the statistical analyses. CC and SL drafted the manuscript. CC, MS, JM, SD, RF, NC, and MW. All authors contributed to the article and approved the submitted version.
