# Supplementary material for: Soluble immune checkpoints as correlates for HIV persistence and T cell function in people with HIV on antiretroviral therapy
Source: Front Immunol. 2023 Mar 28;14:1123342. doi: 10.3389/fimmu.2023.1123342 (PMC10086427; doi:10.3389/fimmu.2023.1123342)
Supplement: Supplementary file 1 [file DataSheet_1.pdf]

Correlation coefficients for Figure 2

	sPD1	sLAG3	sTIM3	sCTLA4	sPDL1	sPDL2	totalDNA	LTR	IntDNA	usRNA	Age
sPD1	1	-0.0592	0.6279	0.0181	-0.0444	0.6308	-0.3469	0.0125	-0.2507	-0.2954	-0.2804
sLAG3	-0.0592	1	-0.4243	0.7414	0.2685	-0.2177	0.5875	-0.0187	0.4629	0.1707	0.3697
sTIM3	0.6279	-0.4243	1	-0.2776	-0.0103	0.7535	-0.6063	-0.2111	-0.4093	-0.416	-0.2794
sCTLA4	0.0181	0.7414	-0.2776	1	0.4419	-0.2802	0.4974	-0.1036	0.3278	0.1064	0.3699
sPDL1	-0.0444	0.2685	-0.0103	0.4419	1	-0.1167	0.5555	-0.2353	0.4688	0.2548	0.1278
sPDL2	0.6308	-0.2177	0.7535	-0.2802	-0.1167	1	-0.4368	-0.1642	-0.3135	-0.2893	-0.2169
totalDNA	-0.3469	0.5875	-0.6063	0.4974	0.5555	-0.4368	1	0.1998	0.8548	0.5823	0.2371
LTR	0.0125	-0.0187	-0.2111	-0.1036	-0.2353	-0.1642	0.1998	1	0.225	0.2614	-0.116
IntDNA	-0.2507	0.4629	-0.4093	0.3278	0.4688	-0.3135	0.8548	0.225	1	0.6192	0.1268
usRNA	-0.2954	0.1707	-0.416	0.1064	0.2548	-0.2893	0.5823	0.2614	0.6192	1	-0.0668
Age	-0.2804	0.3697	-0.2794	0.3699	0.1278	-0.2169	0.2371	-0.116	0.1268	-0.0668	1
ARV_years	-0.0124	0.1124	0.1958	-0.0825	0.4488	0.0618	-0.0218	-0.0842	0.1371	-0.0197	0.0389
CD4count	0.2684	-0.0265	0.1241	-0.0073	-0.1167	0.1575	-0.1547	-0.0604	-0.3822	-0.1808	0.1198
mPD1_CD4	0.2783	-0.4645	0.4479	-0.3782	-0.2428	0.3625	-0.3822	0.172	-0.2148	-0.2459	-0.2083
mLAG3_CD4	0.035	0.1438	0.2476	0.2319	0.2618	0.1822	-0.136	-0.0082	0.284	-0.1372	0.0475
mTIM3_CD4	0.1171	0.067	0.0748	0.241	-0.1056	-0.0269	0.1742	-0.0082	0.1868	0.1521	-0.0341
mCTLA4_CD4	0.0186	0.3125	-0.0579	0.0912	-0.2024	-0.2191	-0.0838	0.1833	0.1587	0.1788	0.1148
mPDL1_CD4	0.0112	0.0806	-0.1326	-0.0611	-0.0485	-0.0181	0.0912	0.0336	0.2833	0.1655	-0.1286
mPDL2_CD4	0.2267	0.2353	0.1378	0.2376	0.2354	0.0223	0.0263	0.1982	0.1835	-0.1009	0.2853
mPD1_CD8	0.4147	-0.4337	0.3972	-0.3444	0.0372	0.3951	-0.306	0.2382	-0.176	-0.1216	-0.2459
mLAG3_CD8	0.1647	0.2225	0.1933	0.1973	0.121	0.2799	-0.1882	-0.072	0.0606	-0.1127	0.2345
mTIM3_CD8	0.4054	0.1603	0.1273	0.2196	0.1293	0.1203	0.0451	0.3085	-0.11	0.0989	-0.287
mCTLA4_CD8	0.0271	0.0615	-0.0901	0.0975	-0.242	-0.0313	-0.2253	0.0924	-0.0671	-0.0048	-0.1932
mPDL1_CD8	-0.1048	0.0136	-0.2248	-0.1941	-0.0968	-0.1131	0.117	0.0931	0.2438	0.3013	-0.1463
mPDL2_CD8	0.0571	0.0749	0.2971	0.1768	0.2926	0.0297	0.0723	0.0674	0.2509	-0.1501	0.246
Ki67_CD4	0.0116	-0.0152	-0.0205	0.1339	-0.0198	-0.305	-0.1082	0.0946	-0.0019	-0.0637	0.0465
HLA-DR_CD4	-0.1121	-0.0694	-0.0941	-0.0532	-0.1408	-0.2196	-0.0105	0.0059	0.2194	-0.2377	0.1164
CD38_CD4	0.2318	0.0982	0.0193	0.0308	0.0815	0.0957	-0.1329	-0.0304	-0.3335	-0.0104	0.1057
HLA-DR_CD3	0.0016	-0.0438	0.0438	-0.1021	-0.189	0.0111	-0.0858	-0.0884	0.0892	-0.0969	0.0164
Ki67_CD8	0.1285	0.0824	0.0852	0.2423	0.407	-0.2084	0.074	-0.0019	-0.0106	-0.1478	-0.0293
HLA-DR_CD8	0.061	0.0828	0.0454	0.0604	0.231	-0.0183	0.1779	0.0696	0.3185	-0.1817	0.0325
CD38_CD8	0.2949	0.0117	0.1987	0.1326	0.489	0.2575	0.0435	-0.0215	-0.1226	-0.0299	0.1327
HLA-DR_CD3	0.2736	-0.1583	0.3442	-0.1213	0.0579	0.2371	-0.1998	-0.0227	-0.0502	-0.2888	-0.1659

	ARV_years	CD4count	mPD1_CD4	mLAG3_CD4	mTIM3_CD4	mCTLA4_CD4	mPDL1_CD4	mPDL2_CD4	mPD1_CD8	mLAG3_CD8	mTIM3_CD8	mCTLA4_CD8
sPD1	-0.0124	0.2684	0.2783	0.035	0.1171	0.0186	0.0112	0.2267	0.4147	0.1647	0.4054	0.0271
sLAG3	0.1124	-0.0265	-0.4645	0.1438	0.067	0.3125	0.0806	0.2353	-0.4337	0.2225	0.1603	0.0615
sTIM3	0.1958	0.1241	0.4479	0.2476	0.0748	-0.0579	-0.1326	0.1378	0.3972	0.1933	0.1273	-0.0901
sCTLA4	-0.0825	-0.0073	-0.3782	0.2319	0.241	0.0912	-0.0611	0.2376	-0.3444	0.1973	0.2196	0.0975
sPDL1	0.4488	-0.1167	-0.2428	0.2618	-0.1056	-0.2024	-0.0485	0.2354	0.0372	0.121	0.1293	-0.242
sPDL2	0.0618	0.1575	0.3625	0.1822	-0.0269	-0.2191	-0.0181	0.0223	0.3951	0.2799	0.1203	-0.0313
totalDNA	-0.0218	-0.1547	-0.3822	-0.136	0.1742	-0.0838	0.0912	0.0263	-0.306	-0.1882	0.0451	-0.2253
LTR	-0.0842	-0.0604	0.172	-0.0082	-0.0082	0.1833	0.0336	0.1982	0.2382	-0.072	0.3085	0.0924
IntDNA	0.1371	-0.3822	-0.2148	0.284	0.1868	0.1587	0.2833	0.1835	-0.176	0.0606	-0.11	-0.0671
usRNA	-0.0197	-0.1808	-0.2459	-0.1372	0.1521	0.1788	0.1655	-0.1009	-0.1216	-0.1127	0.0989	-0.0048
Age	0.0389	0.1198	-0.2083	0.0475	-0.0341	0.1148	-0.1286	0.2853	-0.2459	0.2345	-0.287	-0.1932
ARV_years	1	-0.0682	-0.0213	0.2515	-0.3674	0.1625	0.005	-0.0031	0.1694	0.2128	-0.1254	0.0569
CD4count	-0.0682	1	-0.3734	-0.5055	-0.1081	-0.227	-0.2901	-0.3707	-0.1244	-0.0993	0.1404	-0.3037
mPD1_CD4	-0.0213	-0.3734	1	0.356	0.3203	0.2763	0.4698	0.5172	0.6899	0.0513	0.2441	0.1042
mLAG3_CD4	0.2515	-0.5055	0.356	1	0.0042	0.3706	0.3089	0.4652	0.168	0.6116	-0.1625	0.3132
mTIM3_CD4	-0.3674	-0.1081	0.3203	0.0042	1	0.1535	0.2061	0.1767	0.2944	-0.1254	0.5162	-0.0144
mCTLA4_CD4	0.1625	-0.227	0.2763	0.3706	0.1535	1	0.1909	0.4546	0.2429	0.1449	0.052	0.1984
mPDL1_CD4	0.005	-0.2901	0.4698	0.3089	0.2061	0.1909	1	0.2956	0.1807	0.2363	-0.0728	0.1851
mPDL2_CD4	-0.0031	-0.3707	0.5172	0.4652	0.1767	0.4546	0.2956	1	0.1726	0.3328	0.0067	0.3232
mPD1_CD8	0.1694	-0.1244	0.6899	0.168	0.2944	0.2429	0.1807	0.1726	1	0.0041	0.5643	0.2615
mLAG3_CD8	0.2128	-0.0993	0.0513	0.6116	-0.1254	0.1449	0.2363	0.3328	0.0041	1	-0.1443	0.3426
mTIM3_CD8	-0.1254	0.1404	0.2441	-0.1625	0.5162	0.052	-0.0728	0.0067	0.5643	-0.1443	1	0.1315
mCTLA4_CD8	0.0569	-0.3037	0.1042	0.3132	-0.0144	0.1984	0.1851	0.3232	0.2615	0.3426	0.1315	1
mPDL1_CD8	-0.0084	-0.0985	0.2372	0.0256	0.0987	0.1765	0.7978	0.1511	0.0954	0.2255	-0.0586	0.2234
mPDL2_CD8	0.0447	-0.4144	0.2457	0.3256	0.0839	0.2166	0.0281	0.5758	-0.006	-0.0269	-0.1529	-0.0392
Ki67_CD4	-0.1264	-0.2086	0.3567	0.1222	0.4328	0.3184	0.1045	0.436	0.1911	-0.1665	0.1846	0.1433
HLA-DR_CD4	-0.0272	-0.4393	0.5139	0.2593	0.2492	0.2412	0.4509	0.447	0.2541	0.0059	-0.1725	0.1679
CD38_CD4	0.0559	0.467	-0.5328	-0.4717	-0.2055	-0.1136	-0.5309	-0.2527	-0.2508	-0.184	0.1129	-0.1772
HLA-DR_CD3	0.0113	-0.4771	0.4678	0.2546	0.2826	0.2645	0.4934	0.4354	0.2779	-0.0276	-0.1991	0.1197
Ki67_CD8	-0.0198	0.093	0.2036	-0.1223	0.3268	0.0637	0.0452	0.1388	0.1883	-0.3017	0.2335	-0.0522
HLA-DR_CD8	0.1095	-0.2678	0.1904	0.0817	0.0806	0.0888	0.2317	0.2283	0.2217	0.0448	-0.1272	0.1232
CD38_CD8	0.0342	0.2153	-0.0701	-0.1938	-0.0811	-0.3139	-0.1484	-0.0112	-0.0609	0.0884	0.2162	-0.0939
HLA-DR_CD3	0.1473	-0.1666	0.3616	0.0669	0.0909	0.0838	0.2346	0.2612	0.3781	0.0908	-0.0609	0.0885

	mPDL1_CD8	mPDL2_CD8	Ki67_CD4	HLA-DR_CD4	CD38_CD4	HLA-DR_CD3	Ki67_CD8	HLA-DR_CD8	CD38_CD8	HLA-DR_CD38_CD8
sPD1	-0.1048	0.0571	0.0116	-0.1121	0.2318	0.0016	0.1285	0.061	0.2949	0.2736
sLAG3	0.0136	0.0749	-0.0152	-0.0694	0.0982	-0.0438	0.0824	0.0828	0.0117	-0.1583
sTIM3	-0.2248	0.2971	-0.0205	-0.0941	0.0193	0.0438	0.0852	0.0454	0.1987	0.3442
sCTLA4	-0.1941	0.1768	0.1339	-0.0532	0.0308	-0.1021	0.2423	0.0604	0.1326	-0.1213
sPDL1	-0.0968	0.2926	-0.0198	-0.1408	0.0815	-0.189	0.407	0.231	0.489	0.0579
sPDL2	-0.1131	0.0297	-0.305	-0.2196	0.0957	0.0111	-0.2084	-0.0183	0.2575	0.2371
totalDNA	0.117	0.0723	-0.1082	-0.0105	-0.1329	-0.0858	0.074	0.1779	0.0435	-0.1998
LTR	0.0931	0.0674	0.0946	0.0059	-0.0304	-0.0884	-0.0019	0.0696	-0.0215	-0.0227
IntDNA	0.2438	0.2509	-0.0019	0.2194	-0.3335	0.0892	-0.0106	0.3185	-0.1226	-0.0502
usRNA	0.3013	-0.1501	-0.0637	-0.2377	-0.0104	-0.0969	-0.1478	-0.1817	-0.0299	-0.2888
Age	-0.1463	0.246	0.0465	0.1164	0.1057	0.0164	-0.0293	0.0325	0.1327	-0.1659
ARV_years	-0.0084	0.0447	-0.1264	-0.0272	0.0559	0.0113	-0.0198	0.1095	0.0342	0.1473
CD4count	-0.0985	-0.4144	-0.2086	-0.4393	0.467	-0.4771	0.093	-0.2678	0.2153	-0.1666
mPD1_CD4	0.2372	0.2457	0.3567	0.5139	-0.5328	0.4678	0.2036	0.1904	-0.0701	0.3616
mLAG3_CD4	0.0256	0.3256	0.1222	0.2593	-0.4717	0.2546	-0.1223	0.0817	-0.1938	0.0669
mTIM3_CD4	0.0987	0.0839	0.4328	0.2492	-0.2055	0.2826	0.3268	0.0806	-0.0811	0.0909
mCTLA4_CD4	0.1765	0.2166	0.3184	0.2412	-0.1136	0.2645	0.0637	0.0888	-0.3139	0.0838
mPDL1_CD4	0.7978	0.0281	0.1045	0.4509	-0.5309	0.4934	0.0452	0.2317	-0.1484	0.2346
mPDL2_CD4	0.1511	0.5758	0.436	0.447	-0.2527	0.4354	0.1388	0.2283	-0.0112	0.2612
mPD1_CD8	0.0954	-0.006	0.1911	0.2541	-0.2508	0.2779	0.1883	0.2217	-0.0609	0.3781
mLAG3_CD8	0.2255	-0.0269	-0.1665	0.0059	-0.184	-0.0276	-0.3017	0.0448	0.0884	0.0908
mTIM3_CD8	-0.0586	-0.1529	0.1846	-0.1725	0.1129	-0.1991	0.2335	-0.1272	0.2162	-0.0609
mCTLA4_CD8	0.2234	-0.0392	0.1433	0.1679	-0.1772	0.1197	-0.0522	0.1232	-0.0939	0.0885
mPDL1_CD8	1	-0.0625	-0.01	0.2607	-0.3108	0.225	-0.0628	0.1975	-0.1486	0.1943
mPDL2_CD8	-0.0625	1	0.2927	0.3243	-0.0417	0.333	0.1223	0.2696	-0.0264	0.2603
Ki67_CD4	-0.01	0.2927	1	0.5108	-0.0984	0.5347	0.7298	0.1537	-0.0016	0.2163
HLA-DR_CD4	0.2607	0.3243	0.5108	1	-0.4712	0.9471	0.3195	0.6702	-0.2775	0.6343
CD38_CD4	-0.3108	-0.0417	-0.0984	-0.4712	1	-0.3681	0.0776	-0.1606	0.5974	-0.096
HLA-DR_CD3	0.225	0.333	0.5347	0.9471	-0.3681	1	0.3562	0.5915	-0.173	0.5682
Ki67_CD8	-0.0628	0.1223	0.7298	0.3195	0.0776	0.3562	1	0.1814	0.1609	0.2848
HLA-DR_CD8	0.1975	0.2696	0.1537	0.6702	-0.1606	0.5915	0.1814	1	-0.0607	0.9522
CD38_CD8	-0.1486	-0.0264	-0.0016	-0.2775	0.5974	-0.173	0.1609	-0.0607	1	0.1045
HLA-DR_CD3	0.1943	0.2603	0.2163	0.6343	-0.096	0.5682	0.2848	0.9522	0.1045	1

# p values for Figure 2

	sPD1	sLAG3	sTIM3	sCTLA4	sPDL1	sPDL2	totalDNA	LTR	IntDNA	usRNA	Age	ARV_years	CD4count	mPD1_CD4	mLAG3_CD4	mTIM3_CD4
sPD1	0	0.8897	0.0168	0.3127	0.5381	0	0.098	0.6037	0.2365	0.8115	0.0563	0.9798	0.5723	0.058	0.6658	0.4688
sLAG3	0.8897	0	0.0282	0	0.3511	0.9009	0.0024	0.6605	0.0233	0.6632	0.0018	0.7562	0.5139	8.00E-04	0.2856	0.5499
sTIM3	0.0168	0.0282	0	0.1236	0	0.0143	0.0044	0.5714	0.0331	0.7306	0.0264	0.4327	0.0444	0.2105	0.9527	0.6974
sCTLA4	0.3127	0	0.1236	0	0.2115	0.2545	0.1064	0.6395	0.6871	0.4658	0.0147	0.8462	0.592	0.0095	0.1248	0.3799
sPDL1	0.5381	0.3511	0	0.2115	0	0.915	0.8825	0.4828	0.8614	0.4581	0.1875	0.2599	0.7621	0.6008	0.6949	0.4877
sPDL2	0	0.9009	0.0143	0.2545	0.915	0	0.0386	0.8481	0.1207	0.9659	0.1472	0.5046	0.347	0.0117	0.1259	0.8684
totalDNA	0.098	0.0024	0.0044	0.1064	0.8825	0.0386	0	0.031	0	0.1585	0.135	0.8237	0.0958	0.5154	0.5964	0.246
LTR	0.6037	0.6605	0.5714	0.6395	0.4828	0.8481	0.031	0	0.3254	0.0014	0.3807	0.6719	0.6001	0.976	0.9653	0.191
IntDNA	0.2365	0.0233	0.0331	0.6871	0.8614	0.1207	0	0.3254	0	0.0306	0.6011	0.6052	0.0054	0.9316	0.2162	0.3162
usRNA	0.8115	0.6632	0.7306	0.4658	0.4581	0.9659	0.1585	0.0014	0.0306	0	0.6327	0.8934	0.4924	0.0913	0.7555	0.238
Age	0.0563	0.0018	0.0264	0.0147	0.1875	0.1472	0.135	0.3807	0.6011	0.6327	0	0.753	0.256	0.2874	0.7041	0.7202
ARV_years	0.9798	0.7562	0.4327	0.8462	0.2599	0.5046	0.8237	0.6719	0.6052	0.8934	0.753	0	0.4688	0.3566	0.1273	0.006
CD4count	0.5723	0.5139	0.0444	0.592	0.7621	0.347	0.0958	0.6001	0.0054	0.4924	0.256	0.4688	0	0.0277	9.00E-04	0.7916
mPD1_CD4	0.058	8.00E-04	0.2105	0.0095	0.6008	0.0117	0.5154	0.976	0.9316	0.0913	0.2874	0.3566	0.0277	0	0.1069	0.0029
mLAG3_CD4	0.6658	0.2856	0.9527	0.1248	0.6949	0.1259	0.5964	0.9653	0.2162	0.7555	0.7041	0.1273	9.00E-04	0.1069	0	0.9227
mTIM3_CD4	0.4688	0.5499	0.6974	0.3799	0.4877	0.8684	0.246	0.191	0.3162	0.238	0.7202	0.006	0.7916	0.0029	0.9227	0
mCTLA4_CD4	0.5127	0.1944	0.353	0.592	0.1386	0.1408	0.3706	0.987	0.025	0.9636	0.6818	0.3825	0.0727	0.962	0.0176	0.401
mPDL1_CD4	0.8697	0.9613	0.455	0.9678	0.4153	0.5788	0.4011	0.7963	0.2116	0.4276	0.0773	0.3947	0.5258	0.0031	0.3331	0.0832
mPDL2_CD4	0.6698	0.4452	0.6828	0.8146	0.5102	0.9182	0.1146	0.9258	0.0046	0.2118	0.504	0.9223	0.023	0.0356	0.0116	0.0668
mPD1_CD8	0.0076	2.00E-04	0.0435	0.0056	0.7436	7.00E-04	0.035	0.0981	0.0602	0.0208	0.0986	0.3455	0.4055	0	0.5825	0.0722
mLAG3_CD8	0.4032	0.184	0.4268	0.2742	0.464	0.0623	0.1379	0.5914	0.7761	0.7468	0.0711	0.1482	0.5827	0.7617	0	0.9488
mTIM3_CD8	0.0289	0.2602	0.8393	0.6588	0.5682	0.1418	0.813	0.0053	0.381	0.0786	0.0887	0.2893	0.2896	0.4449	0.594	0.0012
mCTLA4_CD8	0.6814	0.7258	0.7473	0.8895	0.8073	0.9057	0.2821	0.3442	0.4996	0.3952	0.1076	0.267	0.0616	0.7171	0.0704	0.6217
mPDL1_CD8	0.8428	0.7602	0.2066	0.6323	0.1299	0.471	0.3402	0.8057	0.2071	0.2227	0.3056	0.9975	0.7313	0.1258	0.8552	0.4225
mPDL2_CD8	0.8551	0.6921	0.869	0.7447	0.7422	0.7539	0.4371	0.9355	0.0117	0.3938	0.3565	0.6353	0.0772	0.6834	0.0777	0.3761
Ki67_CD4	0.4079	0.6103	0.6268	0.6988	0.7561	0.3219	0.678	0.7277	0.2162	0.556	0.6491	0.6597	0.1647	0.1318	0.3029	0.0637
HLA-DR_CD4	0.8473	0.824	0.6925	0.5778	0.5487	0.6707	0.0242	0.3665	0.0258	0.198	0.2845	0.5327	0.0175	0	0.159	0.0165
CD38_CD4	0.0894	0.3818	0.456	0.3583	0.9755	0.3256	0.3673	0.3797	0.0519	0.8695	0.6391	0.8265	0.0104	3.00E-04	0.0054	0.1218
HLA-DR_CD3	0.8824	0.9333	0.5461	0.6411	0.6248	0.6676	0.1717	0.2778	0.0399	0.644	0.3631	0.8534	4.00E-04	0	0.1383	0.0144
Ki67_CD8	0.5842	0.5891	0.9457	0.9334	0.2886	0.4671	0.9217	0.9218	0.6971	0.7907	0.4695	0.4424	0.7488	0.4127	0.89	0.2152
HLA-DR_CD8	0.5612	0.4704	0.9841	0.5634	0.7641	0.8772	0.0179	0.7721	0.0911	0.3656	0.4539	0.8731	0.0337	0.0039	0.8124	0.2688
CD38_CD8	0.0135	0.3479	0.3593	0.1797	0.3772	0.0465	0.5741	0.541	0.5483	0.8114	0.3808	0.5237	0.278	0.8656	0.4866	0.563
HLA-DR_CD3	0.1047	0.3072	0.3084	0.9232	0.9499	0.0176	0.6186	0.7515	0.7059	0.3612	0.6637	0.6166	0.2704	0.0019	0.8971	0.2871

	mCTLA4_CD4	mPDL1_CD4	mPDL2_CD4	mPD1_CD8	mLAG3_CD8	mTIM3_CD8	mCTLA4_CD8	mPDL1_CD8	mPDL2_CD8	Ki67_CD4	HLA-DR_CD4	CD38_CD4	HLA-DR_CD3	Ki67_CD8	HLA-DR_CD8	CD38_CD8	HLA-DR_CD3
sPD1	0.5127	0.8697	0.6698	0.0076	0.4032	0.0289	0.6814	0.8428	0.8551	0.4079	0.8473	0.0894	0.8824	0.5842	0.5612	0.0135	0.1047
sLAG3	0.1944	0.9613	0.4452	2.00E-04	0.184	0.2602	0.7258	0.7602	0.6921	0.6103	0.824	0.3818	0.9333	0.5891	0.4704	0.3479	0.3072
sTIM3	0.353	0.455	0.6828	0.0435	0.4268	0.8393	0.7473	0.2066	0.869	0.6268	0.6925	0.456	0.5461	0.9457	0.9841	0.3593	0.3084
sCTLA4	0.592	0.9678	0.8146	0.0056	0.2742	0.6588	0.8895	0.6323	0.7447	0.6988	0.5778	0.3583	0.6411	0.9334	0.5634	0.1797	0.9232
sPDL1	0.1386	0.4153	0.5102	0.7436	0.464	0.5682	0.8073	0.1299	0.7422	0.7561	0.5487	0.9755	0.6248	0.2886	0.7641	0.3772	0.9499
sPDL2	0.1408	0.5788	0.9182	7.00E-04	0.0623	0.1418	0.9057	0.471	0.7539	0.3219	0.6707	0.3256	0.6676	0.4671	0.8772	0.0465	0.0176
totalDNA	0.3706	0.4011	0.1146	0.035	0.1379	0.813	0.2821	0.3402	0.4371	0.678	0.0242	0.3673	0.1717	0.9217	0.0179	0.5741	0.6186
LTR	0.987	0.7963	0.9258	0.0981	0.5914	0.0053	0.3442	0.8057	0.9355	0.7277	0.3665	0.3797	0.2778	0.9218	0.7721	0.541	0.7515
IntDNA	0.025	0.2116	0.0046	0.0602	0.7761	0.381	0.4996	0.2071	0.0117	0.2162	0.0258	0.0519	0.0399	0.6971	0.0911	0.5483	0.7059
usRNA	0.9636	0.4276	0.2118	0.0208	0.7468	0.0786	0.3952	0.2227	0.3938	0.556	0.198	0.8695	0.644	0.7907	0.3656	0.8114	0.3612
Age	0.6818	0.0773	0.504	0.0986	0.0711	0.0887	0.1076	0.3056	0.3565	0.6491	0.2845	0.6391	0.3631	0.4695	0.4539	0.3808	0.6637
ARV_years	0.3825	0.3947	0.9223	0.3455	0.1482	0.2893	0.267	0.9975	0.6353	0.6597	0.5327	0.8265	0.8534	0.4424	0.8731	0.5237	0.6166
CD4count	0.0727	0.5258	0.023	0.4055	0.5827	0.2896	0.0616	0.7313	0.0772	0.1647	0.0175	0.0104	4.00E-04	0.7488	0.0337	0.278	0.2704
mPD1_CD4	0.962	0.0031	0.0356	0	0.7617	0.4449	0.7171	0.1258	0.6834	0.1318	0	3.00E-04	0	0.4127	0.0039	0.8656	0.0019
mLAG3_CD4	0.0176	0.3331	0.0116	0.5825	0	0.594	0.0704	0.8552	0.0777	0.3029	0.159	0.0054	0.1383	0.89	0.8124	0.4866	0.8971
mTIM3_CD4	0.401	0.0832	0.0668	0.0722	0.9488	0.0012	0.6217	0.4225	0.3761	0.0637	0.0165	0.1218	0.0144	0.2152	0.2688	0.563	0.2871
mCTLA4_CD4	0	0.5085	0	0.6519	0.0702	0.6523	0.5584	0.5175	0	3.00E-04	0.6434	0.5696	0.1804	0.1298	0.3103	0.1669	0.2628
mPDL1_CD4	0.5085	0	0.2578	0.363	0.2359	0.9995	0.5539	0	0.791	0.9571	0.168	0.0013	0.1235	0.5861	0.4646	0.9544	0.6213
mPDL2_CD4	0	0.2578	0	0.5984	0.0133	0.8912	0.577	0.3503	0	5.00E-04	0.0108	0.3386	0.0053	0.2424	0.0243	0.9354	0.0294
mPD1_CD8	0.6519	0.363	0.5984	0	0.9993	0	0.0249	0.6592	0.6297	0.2418	0.628	0.0316	0.0879	0.1914	0.4004	0.7828	0.0036
mLAG3_CD8	0.0702	0.2359	0.0133	0.9993	0	0.9757	0.0542	0.0859	0.2114	0.7219	0.6451	0.1956	0.9228	0.1961	0.8466	0.4698	0.9596
mTIM3_CD8	0.6523	0.9995	0.8912	0	0.9757	0	0.3483	0.8822	0.3021	0.9123	0.1844	0.9914	0.1774	0.8736	0.2993	0.2438	0.5784
mCTLA4_CD8	0.5584	0.5539	0.577	0.0249	0.0542	0.3483	0	0.6649	0.6991	0.7607	0.9991	0.2389	0.991	0.2627	0.8582	0.2741	0.8085
mPDL1_CD8	0.5175	0	0.3503	0.6592	0.0859	0.8822	0.6649	0	0.8335	0.8251	0.6913	0.0587	0.6851	0.4834	0.5503	0.621	0.4913
mPDL2_CD8	0	0.791	0	0.6297	0.2114	0.3021	0.6991	0.8335	0	3.00E-04	0.1935	0.9433	0.0622	0.0282	0.2066	0.8671	0.1446
Ki67_CD4	3.00E-04	0.9571	5.00E-04	0.2418	0.7219	0.9123	0.7607	0.8251	3.00E-04	0	0.0407	0.8244	8.00E-04	0	0.0723	0.9213	0.0086
HLA-DR_CD4	0.6434	0.168	0.0108	0.628	0.6451	0.1844	0.9991	0.6913	0.1935	0.0407	0	0.0033	0	0.1236	0	0.4469	0
CD38_CD4	0.5696	0.0013	0.3386	0.0316	0.1956	0.9914	0.2389	0.0587	0.9433	0.8244	0.0033	0	0.0146	0.787	0.2116	0	0.598
HLA-DR_CD3	0.1804	0.1235	0.0053	0.0879	0.9228	0.1774	0.991	0.6851	0.0622	8.00E-04	0	0.0146	0	0.0052	0	0.5965	0
Ki67_CD8	0.1298	0.5861	0.2424	0.1914	0.1961	0.8736	0.2627	0.4834	0.0282	0	0.1236	0.787	0.0052	0	0.0921	0.4592	0.0046
HLA-DR_CD8	0.3103	0.4646	0.0243	0.4004	0.8466	0.2993	0.8582	0.5503	0.2066	0.0723	0	0.2116	0	0.0921	0	0.9915	0
CD38_CD8	0.1669	0.9544	0.9354	0.7828	0.4698	0.2438	0.2741	0.621	0.8671	0.9213	0.4469	0	0.5965	0.4592	0.9915	0	0.2795
HLA-DR_CD3	0.2628	0.6213	0.0294	0.0036	0.9596	0.5784	0.8085	0.4913	0.1446	0.0086	0	0.598	0	0.0046	0	0.2795	0

Correlation coefficient for Figure 3

	totalDNA	IntDNA	sPD1	sLAG3	sTIM3	sCTLA4	sPDL1	sPDL2	Gag_CD4_CD	Gag_CD4_IFI	Gag_CD4_IL2	Gag_CD4_TN
totalDNA	1	0.7383	0.0811	0.0313	0.0888	0.0011	0.641	0.0103	-0.2288	-0.0203	0.1398	0.1373
IntDNA	0.7383	1	-0.0083	0.0294	0.1382	-0.0403	0.4048	-0.0301	-0.0978	0.1115	0.0888	0.0492
sPD1	0.0811	-0.0083	1	0.6582	0.5407	0.6791	0.1672	0.552	-0.3542	0.022	0.1164	0.2989
sLAG3	0.0313	0.0294	0.6582	1	0.5969	0.6272	0.2112	0.5284	-0.5728	2.00E-04	0.1403	0.1998
sTIM3	0.0888	0.1382	0.5407	0.5969	1	0.5766	0.1188	0.7011	-0.2717	0.1407	0.3156	0.3029
sCTLA4	0.0011	-0.0403	0.6791	0.6272	0.5766	1	0.5105	0.3179	-0.1683	0.0067	0.1751	0.23
sPDL1	0.641	0.4048	0.1672	0.2112	0.1188	0.5105	1	0.0638	-0.0528	0.469	0.4064	0.6879
sPDL2	0.0103	-0.0301	0.552	0.5284	0.7011	0.3179	0.0638	1	-0.3294	0.1157	0.0639	0.2836
Gag_CD4_CD	-0.2288	-0.0978	-0.3542	-0.5728	-0.2717	-0.1683	-0.0528	-0.3294	1	0.1621	0.1403	-0.2047
Gag_CD4_IFI	-0.0203	0.1115	0.022	2.00E-04	0.1407	0.0067	0.469	0.1157	0.1621	1	0.5546	0.3854
Gag_CD4_IL2	0.1398	0.0888	0.1164	0.1403	0.3156	0.1751	0.4064	0.0639	0.1403	0.5546	1	0.439
Gag_CD4_TN	0.1373	0.0492	0.2989	0.1998	0.3029	0.23	0.6879	0.2836	-0.2047	0.3854	0.439	1
Gag_CD8_CD	0.0896	0.0632	0.2272	0.2703	0.3633	0.1601	-0.5434	0.493	-0.1141	-0.0374	0.1194	0.079
Gag_CD8_IFI	-0.3202	-0.1605	0.1603	0.1785	0.2188	-7.00E-04	-0.5434	0.4185	6.00E-04	0.0779	0.0846	0.1765
Gag_CD8_IL2	-0.4737	-0.3018	-0.1966	-0.0348	0.0483	-0.069	-0.411	0.1968	0.2182	0.3871	0.1667	-0.0198
Gag_CD8_TN	-0.1982	-0.1859	0.2014	0.122	0.2442	-0.0126	-0.2968	0.5203	0.044	0.196	0.1266	0.1198
Nef_CD4_CD	0.0311	0.026	0.055	0.0853	0.2902	0.1066	-0.3151	0.1765	0.0997	-0.1072	0.0907	0.0226
Nef_CD4_IFN	-0.1696	0.0061	0.0414	0.0074	0.1212	0.0572	0.2621	0.054	0.4203	0.4591	0.2527	-0.103
Nef_CD4_IL2	0.1873	0.0829	0.3836	0.3365	0.4689	0.4353	0.5652	0.2608	0.008	0.3325	0.6419	0.3364
Nef_CD4_TN	-0.0032	0.0826	0.2323	0.0398	0.148	0.1181	0.3402	0.07	0.1235	0.1298	0.0968	0.2682
Nef_CD8_CD	0.2586	0.1965	0.3371	0.1778	0.0804	0.2692	0.1701	0.0061	-0.0451	0.0268	0.0118	0.0199
Nef_CD8_IFN	-0.1377	-0.0474	0.1491	0.2071	0.0759	0.1248	0.2028	0.1996	-0.014	0.173	0.0883	0.0432
Nef_CD8_IL2	-0.189	-0.1928	0.3566	0.2302	0.2504	0.3109	-0.1471	0.1614	0.0157	0.0707	0.31	0.0397
Nef_CD8_TN	-0.0831	0.0198	0.0304	-0.0109	0.0138	0.0396	0.2055	0.0957	0.1419	0.198	0.1351	-0.0521

	Gag_CD8_CD	Gag_CD8_IFI	Gag_CD8_IL2	Gag_CD8_TN	Nef_CD4_CD	Nef_CD4_IFN	Nef_CD4_IL2	Nef_CD4_TN	Nef_CD8_CD	Nef_CD8_IFN	Nef_CD8_IL2	Nef_CD8_TN
totalDNA	0.0896	-0.3202	-0.4737	-0.1982	0.0311	-0.1696	0.1873	-0.0032	0.2586	-0.1377	-0.189	-0.0831
IntDNA	0.0632	-0.1605	-0.3018	-0.1859	0.026	0.0061	0.0829	0.0826	0.1965	-0.0474	-0.1928	0.0198
sPD1	0.2272	0.1603	-0.1966	0.2014	0.055	0.0414	0.3836	0.2323	0.3371	0.1491	0.3566	0.0304
sLAG3	0.2703	0.1785	-0.0348	0.122	0.0853	0.0074	0.3365	0.0398	0.1778	0.2071	0.2302	-0.0109
sTIM3	0.3633	0.2188	0.0483	0.2442	0.2902	0.1212	0.4689	0.148	0.0804	0.0759	0.2504	0.0138
sCTLA4	0.1601	-7.00E-04	-0.069	-0.0126	0.1066	0.0572	0.4353	0.1181	0.2692	0.1248	0.3109	0.0396
sPDL1	-0.5434	-0.5434	-0.411	-0.2968	-0.3151	0.2621	0.5652	0.3402	0.1701	0.2028	-0.1471	0.2055
sPDL2	0.493	0.4185	0.1968	0.5203	0.1765	0.054	0.2608	0.07	0.0061	0.1996	0.1614	0.0957
Gag_CD4_CD	-0.1141	6.00E-04	0.2182	0.044	0.0997	0.4203	0.008	0.1235	-0.0451	-0.014	0.0157	0.1419
Gag_CD4_IFI	-0.0374	0.0779	0.3871	0.196	-0.1072	0.4591	0.3325	0.1298	0.0268	0.173	0.0707	0.198
Gag_CD4_IL2	0.1194	0.0846	0.1667	0.1266	0.0907	0.2527	0.6419	0.0968	0.0118	0.0883	0.31	0.1351
Gag_CD4_TN	0.079	0.1765	-0.0198	0.1198	0.0226	-0.103	0.3364	0.2682	0.0199	0.0432	0.0397	-0.0521
Gag_CD8_CD	1	0.6975	0.3409	0.7209	0.3261	0.1654	0.1611	0.2514	0.3734	0.3831	0.4251	0.3301
Gag_CD8_IFI	0.6975	1	0.4071	0.8254	0.32	0.2372	-0.0267	0.1385	0.077	0.344	0.2689	0.356
Gag_CD8_IL2	0.3409	0.4071	1	0.4863	0.1496	0.251	0.1627	-0.074	-0.0046	0.3109	0.3849	0.4108
Gag_CD8_TN	0.7209	0.8254	0.4863	1	0.1772	0.2948	0.0555	0.2356	0.1885	0.3783	0.236	0.4533
Nef_CD4_CD	0.3261	0.32	0.1496	0.1772	1	0.0138	0.4222	-0.0496	0.0908	0.2663	0.3441	0.2466
Nef_CD4_IFN	0.1654	0.2372	0.251	0.2948	0.0138	1	0.2486	0.3893	0.1433	0.3322	0.2199	0.3979
Nef_CD4_IL2	0.1611	-0.0267	0.1627	0.0555	0.4222	0.2486	1	0.1298	0.2696	0.37	0.4762	0.2564
Nef_CD4_TN	0.2514	0.1385	-0.074	0.2356	-0.0496	0.3893	0.1298	1	0.4619	0.283	0.285	0.2056
Nef_CD8_CD	0.3734	0.077	-0.0046	0.1885	0.0908	0.1433	0.2696	0.4619	1	0.6097	0.512	0.4873
Nef_CD8_IFN	0.3831	0.344	0.3109	0.3783	0.2663	0.3322	0.37	0.283	0.6097	1	0.5482	0.8177
Nef_CD8_IL2	0.4251	0.2689	0.3849	0.236	0.3441	0.2199	0.4762	0.285	0.512	0.5482	1	0.4758
Nef_CD8_TN	0.3301	0.356	0.4108	0.4533	0.2466	0.3979	0.2564	0.2056	0.4873	0.8177	0.4758	1

# p values for Figure 3

	totalDNA	IntDNA	sPD1	sLAG3	sTIM3	sCTLA4	sPDL1	sPDL2	Gag_CD4_CD	Gag_CD4_IF	Gag_CD4_IL	Gag_CD4_TN
totalDNA	0	0	0.7949	0.9235	0.3739	0.8281	0.8941	0.8028	0.1951	0.9572	0.7986	0.432
IntDNA	0	0	0.5164	0.9897	0.4945	0.3952	0.89	0.7132	0.2884	0.9342	0.6538	0.4984
sPD1	0.7949	0.5164	0	0	0.0023	0	0.1588	0	0.0553	0.8928	0.7888	0.0386
sLAG3	0.9235	0.9897	0	0	0.6848	0	0.3461	6.00E-04	0.0104	0.9565	0.5679	0.3206
sTIM3	0.3739	0.4945	0.0023	0.6848	0	9.00E-04	0	0.1135	0.5732	0.6505	0.5645	0.0976
sCTLA4	0.8281	0.3952	0	0	9.00E-04	0	0.1015	0.0233	0.2536	0.5518	0.8984	0.076
sPDL1	0.8941	0.89	0.1588	0.3461	0	0.1015	0	0.4845	0.832	0.5672	0.5534	0.4135
sPDL2	0.8028	0.7132	0	6.00E-04	0.1135	0.0233	0.4845	0	0.1057	0.3851	0.3848	0.0704
Gag_CD4_CD	0.1951	0.2884	0.0553	0.0104	0.5732	0.2536	0.832	0.1057	0	0.629	0.3449	0.5554
Gag_CD4_IF	0.9572	0.9342	0.8928	0.9565	0.6505	0.5518	0.5672	0.3851	0.629	0	6.00E-04	0.0058
Gag_CD4_IL	0.7986	0.6538	0.7888	0.5679	0.5645	0.8984	0.5534	0.3848	0.3449	6.00E-04	0	0.0408
Gag_CD4_TN	0.432	0.4984	0.0386	0.3206	0.0976	0.076	0.4135	0.0704	0.5554	0.0058	0.0408	0
Gag_CD8_CD	0.542	0.7184	0.1935	0.0837	0.5037	0.1829	0.3014	0.0372	0.9952	0.8317	0.3606	0.9136
Gag_CD8_IF	0.0977	0.0948	0.3936	0.2145	0.4826	0.4081	0.2341	0.0258	0.4785	0.6373	0.1519	0.5401
Gag_CD8_IL	0.2323	0.8663	0.0503	0.4005	0.809	0.6031	0.5982	0.3683	0.3091	2.00E-04	0.4116	0.2129
Gag_CD8_TN	0.205	0.1492	0.7656	0.5857	0.674	0.7004	0.3797	0.0046	0.4166	0.4972	0.1294	0.9747
Nef_CD4_CD	0.3755	0.8491	0.4699	0.7345	0.4208	0.8857	0.333	0.2928	0.513	0.2201	0.8718	0.5905
Nef_CD4_IF	0.66	0.9944	0.8296	0.7984	0.7744	0.937	0.9287	0.6938	0.194	7.00E-04	0.0218	0.9824
Nef_CD4_IL2	0.2529	0.7511	0.08	0.0521	0.4452	0.0786	0.9829	0.0863	0.3951	0.1338	6.00E-04	0.0367
Nef_CD4_TN	0.7866	0.9871	0.3808	0.8332	0.9743	0.9191	0.8287	0.817	0.3103	0.6407	0.9907	0.2326
Nef_CD8_CD	0.175	0.2316	0.2359	0.7191	0.9207	0.4732	0.627	0.8141	0.2442	0.8765	0.7279	0.781
Nef_CD8_IF	0.511	0.5492	0.539	0.8609	0.2257	0.5223	0.5532	0.4944	0.1172	0.2095	0.7713	0.4068
Nef_CD8_IL2	0.7231	0.3661	0.6568	0.675	0.8322	0.8852	0.7475	0.9624	0.1801	0.9467	0.1963	0.7855
Nef_CD8_TN	0.815	0.4923	0.9145	0.2976	0.5983	0.8511	0.7704	0.5444	0.1293	0.6743	0.9401	0.9582

	Gag_CD8_CD	Gag_CD8_IF	Gag_CD8_IL	Gag_CD8_TN	Nef_CD4_CD	Nef_CD4_IF	Nef_CD4_IL2	Nef_CD4_TN	Nef_CD8_CD	Nef_CD8_IF	Nef_CD8_IL2	Nef_CD8_TN
totalDNA	0.542	0.0977	0.2323	0.205	0.3755	0.66	0.2529	0.7866	0.175	0.511	0.7231	0.815
IntDNA	0.7184	0.0948	0.8663	0.1492	0.8491	0.9944	0.7511	0.9871	0.2316	0.5492	0.3661	0.4923
sPD1	0.1935	0.3936	0.0503	0.7656	0.4699	0.8296	0.08	0.3808	0.2359	0.539	0.6568	0.9145
sLAG3	0.0837	0.2145	0.4005	0.5857	0.7345	0.7984	0.0521	0.8332	0.7191	0.8609	0.675	0.2976
sTIM3	0.5037	0.4826	0.809	0.674	0.4208	0.7744	0.4452	0.9743	0.9207	0.2257	0.8322	0.5983
sCTLA4	0.1829	0.4081	0.6031	0.7004	0.8857	0.937	0.0786	0.9191	0.4732	0.5223	0.8852	0.8511
sPDL1	0.3014	0.2341	0.5982	0.3797	0.333	0.9287	0.9829	0.8287	0.627	0.5532	0.7475	0.7704
sPDL2	0.0372	0.0258	0.3683	0.0046	0.2928	0.6938	0.0863	0.817	0.8141	0.4944	0.9624	0.5444
Gag_CD4_CD	0.9952	0.4785	0.3091	0.4166	0.513	0.194	0.3951	0.3103	0.2442	0.1172	0.1801	0.1293
Gag_CD4_IF	0.8317	0.6373	2.00E-04	0.4972	0.2201	7.00E-04	0.1338	0.6407	0.8765	0.2095	0.9467	0.6743
Gag_CD4_IL	0.3606	0.1519	0.4116	0.1294	0.8718	0.0218	6.00E-04	0.9907	0.7279	0.7713	0.1963	0.9401
Gag_CD4_TN	0.9136	0.5401	0.2129	0.9747	0.5905	0.9824	0.0367	0.2326	0.781	0.4068	0.7855	0.9582
Gag_CD8_CD	0	0	0.8487	0	0.0011	0.1555	0.0543	0.1905	0.0279	0.034	0.0763	0.2134
Gag_CD8_IF	0	0	0.5743	0	0.0313	0.0844	0.528	0.3818	0.4698	0.0529	0.2241	0.2665
Gag_CD8_IL	0.8487	0.5743	0	0.2224	0.7468	0.125	0.9658	0.4662	0.7281	0.3524	0.8602	0.3644
Gag_CD8_TN	0	0	0.2224	0	0.3871	0.4689	0.8478	0.3939	0.6992	0.309	0.7616	0.3168
Nef_CD4_CD	0.0011	0.0313	0.7468	0.3871	0	0.9984	0.0339	0.7931	0.0691	0.0212	0.025	0.2255
Nef_CD4_IF	0.1555	0.0844	0.125	0.4689	0.9984	0	0.169	0.0043	0.5708	0.0395	0.4627	0.2441
Nef_CD4_IL2	0.0543	0.528	0.9658	0.8478	0.0339	0.169	0	0.5295	0.124	0.0278	0.0018	0.1839
Nef_CD4_TN	0.1905	0.3818	0.4662	0.3939	0.7931	0.0043	0.5295	0	0.001	0.0027	0.0982	0.0015
Nef_CD8_CD	0.0279	0.4698	0.7281	0.6992	0.0691	0.5708	0.124	0.001	0	0	0	0
Nef_CD8_IF	0.034	0.0529	0.3524	0.309	0.0212	0.0395	0.0278	0.0027	0	0	0	0
Nef_CD8_IL2	0.0763	0.2241	0.8602	0.7616	0.025	0.4627	0.0018	0.0982	0	0	0	5.00E-04
Nef_CD8_TN	0.2134	0.2665	0.3644	0.3168	0.2255	0.2441	0.1839	0.0015	0	0	5.00E-04	0
