# Supplementary material for: Soluble immune checkpoints as correlates for HIV persistence and T cell function in people with HIV on antiretroviral therapy
Source: Front Immunol. 2023 Mar 28;14:1123342. doi: 10.3389/fimmu.2023.1123342 (PMC10086427; doi:10.3389/fimmu.2023.1123342)
Supplement: Supplementary file 2 [file Table_1.pdf]

Supplementary Material

Soluble immune checkpoints as correlates for HIV persistence and T cell function in people with HIV on antiretroviral therapy

Chris Y. Chiu[1], Maya D. Schou[1], James H. McMahon[2], Steve G Deeks[3], Rémi Fromentin[4,5], Nicolas Chomont [4,5], Michelle N. Wykes[6], Thomas A. Rasmussen[1,7] Sharon R. Lewin[1,2,8*]

[1] Department of Infectious Diseases, The University of Melbourne at The Peter Doherty Institute for Infection and Immunity, Melbourne, Victoria, Australia

[2] Department of Infectious Diseases, Alfred Hospital and Monash University and the Alfred Hospital, Melbourne, Victoria, Australia

[3] Department of Medicine, University California San Francisco, San Francisco, CA

[4] Centre de Recherche du Centre Hospitalier de l'Université de Montréal, Montreal, Canada

[5] Department of Microbiology, Infectiology and Immunology, Faculty of Medicine, Université de Montréal, Montreal, Canada

[6] QIMR Berghofer Medical Research Institute, 300 Herston Road, Herston, Brisbane, Queensland 4006, Australia

[7] Department of Infectious Diseases, Aarhus University Hospital, Aarhus, Denmark

[8] Victorian Infectious Diseases Service, Royal Melbourne Hospital at the Peter Doherty Institute for Infection and Immunity, Melbourne, Australia

*** Correspondence:** Sharon Lewin: sharon.lewin@unimelb.edu.au

Supplementary Table 1: Median and interquartile range of virological and cellular parameters for on ART participants (SCOPE cohort, n = 48)

Parameter	Median	25th percentile	75th percentile
totalDNA (copies per million CD4+)	1039	526	2039
LTR (copies per million CD4+)	19.5	4	53
IntDNA (copies per million CD4+)	342	156	736.5
usRNA (copies per million CD4+)	23	12	37.5
mPD1_CD4 (%)	12.2	8.78	16.35
mLAG3_CD4 (%)	11.95	8.99	16.05
mTIM3_CD4 (%)	0.81	0.61	1.46
mCTLA4_CD4 (%)	0.73	0.6	0.94
mPDL1_CD4 (%)	2.05	1.46	2.86
mPDL2_CD4 (%)	0.34	0.25	0.49
mPD1_CD8 (%)	13.75	8.57	18.6
mLAG3_CD8 (%)	22.1	14.5	29.3
mTIM3_CD8 (%)	3.08	2.08	4.44
mCTLA4_CD8 (%)	0.64	0.43	0.91
mPDL1_CD8 (%)	5.44	3.03	7.15
mPDL2_CD8 (%)	0.1	0.08	0.14
Ki67_CD4 (%)	1.49	1.19	1.98
HLA-DR_CD4 (%)	6.13	4.69	9.37
CD38_CD4 (%)	48.65	41.5	59.6
HLA-DR_CD38_CD4 (%)	3.84	2.56	6.2
Ki67_CD8 (%)	0.87	0.62	1.15
HLA-DR_CD8 (%)	15.45	10.7	20.7
CD38_CD8 (%)	53.1	47.05	60.15
HLA-DR_CD38_CD8 (%)	9.37	6.33	13.05
